# Jimena: efficient computing and system state identification for genetic regulatory networks

**DOI:** 10.1186/1471-2105-14-306

**Published:** 2013-10-11

**Authors:** Stefan Karl, Thomas Dandekar

**Affiliations:** 1 Department of Bioinformatics, University of Würzburg, Am Hubland, Würzburg, Germany

**Keywords:** Boolean function, Genetic regulatory network, Interpolation, Stable state, Binary decision diagram, Boolean tree

## Abstract

**Background:**

Boolean networks capture switching behavior of many naturally occurring regulatory networks. For semi-quantitative modeling, interpolation between ON and OFF states is necessary. The high degree polynomial interpolation of Boolean genetic regulatory networks (GRNs) in cellular processes such as apoptosis or proliferation allows for the modeling of a wider range of node interactions than continuous activator-inhibitor models, but suffers from scaling problems for networks which contain nodes with more than ~10 inputs. Many GRNs from literature or new gene expression experiments exceed those limitations and a new approach was developed.

**Results:**

(i) As a part of our new GRN simulation framework Jimena we introduce and setup Boolean-tree-based data structures; (ii) corresponding algorithms greatly expedite the calculation of the polynomial interpolation in almost all cases, thereby expanding the range of networks which can be simulated by this model in reasonable time. (iii) Stable states for discrete models are efficiently counted and identified using binary decision diagrams. As application example, we show how system states can now be sampled efficiently in small up to large scale hormone disease networks (*Arabidopsis thaliana* development and immunity, pathogen *Pseudomonas syringae* and modulation by cytokinins and plant hormones).

**Conclusions:**

Jimena simulates currently available GRNs about 10-100 times faster than the previous implementation of the polynomial interpolation model and even greater gains are achieved for large scale-free networks. This speed-up also facilitates a much more thorough sampling of continuous state spaces which may lead to the identification of new stable states. Mutants of large networks can be constructed and analyzed very quickly enabling new insights into network robustness and behavior.

## Background

For the simulation of genetic regulatory networks (GRNs) two important paradigms have been used: Discrete models, where each node has a value of either 0 or 1 and Boolean expressions are used to update the values of the nodes in each simulation step using an updating scheme like CRBN (classical random Boolean networks) or ARBN (asynchronous random Boolean networks) [1], and continuous models where nodes have values in the interval [0,1] and real-valued ODEs (ordinary differential equations) determine the behavior of the network.

Two commonly used continuous modeling paradigms for GRNs are activator-inhibitor-models such as the exponential standardized qualitative dynamical systems model [2], which is implemented in the SQUAD [3] simulation package, and real-valued interpolations of Boolean functions which allow for more complex node interactions.

These interpolations extend the domain and the codomain of Boolean functions {0, 1}^*n*^ → {0, 1} by defining functions [0, 1]^*n*^ → [0, 1]^*n*^ which mimic the behavior of the original function for intermediate input values in the interval (0,1). For example, an adequate interpolation of the function *B* ' (*a*, *b*) = *a* OR *b* for which *B* ' (0, 0) = 0 and *B* ' (0, 1) = 1 would be expected to return a value 0 < ξ <1 for the input (0,0.5).

**Figure 1:**
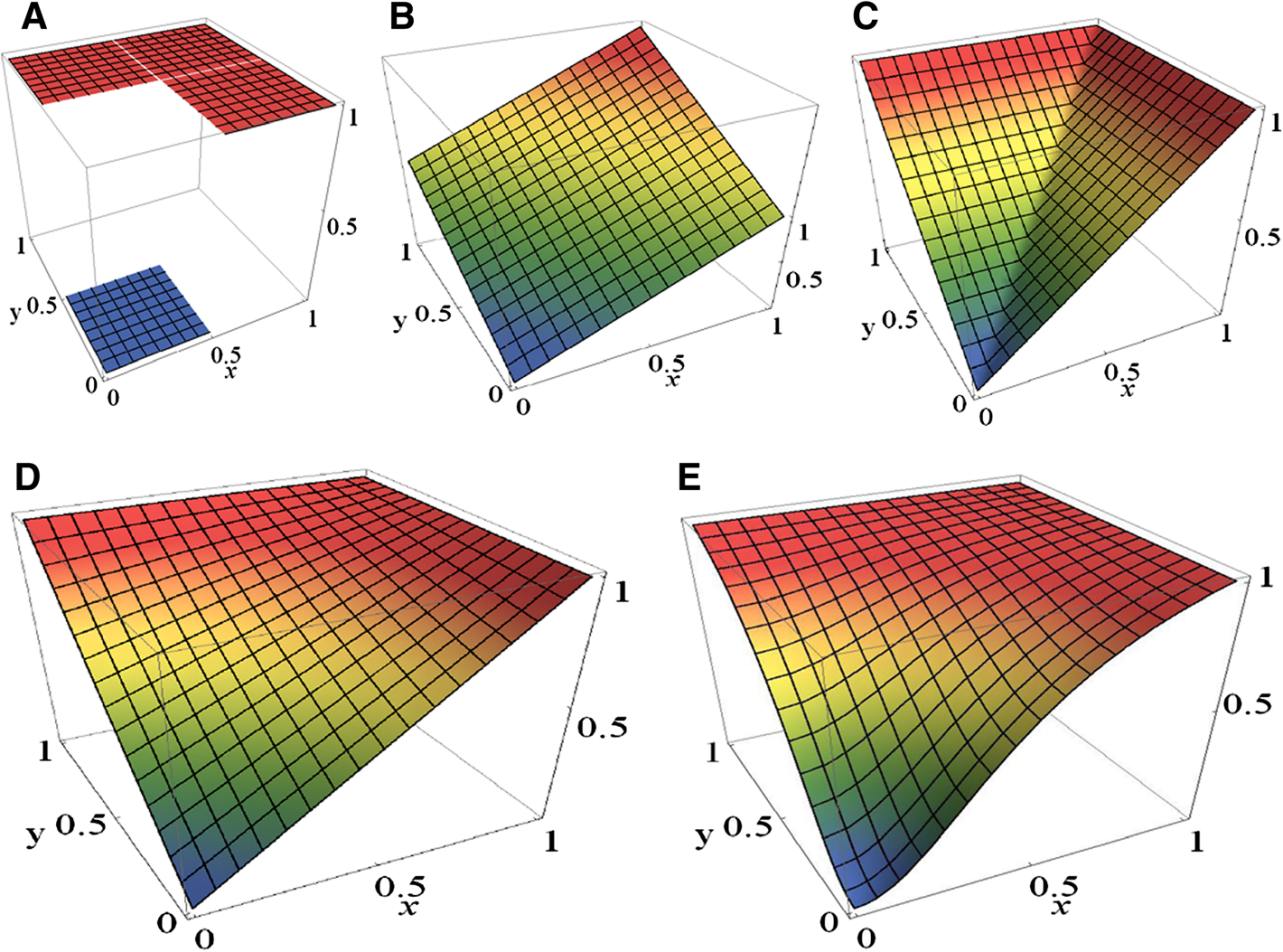
**Interpolations of the Boolean function x OR y.** Different panels show **A)** piecewise linear functions **B)** product-sum fuzzy logic **C)** min-max fuzzy logic **D)** Boole-Cubes **E)** Hill-cubes.

Wittmann et al. [4] reviewed in detail several common interpolation strategies such as min-max fuzzy logic, product-sum fuzzy logic and piecewise linear functions (implemented for example in the BooleanNet simulation package [5]). We illustrate different interpolation functions in Figure 1. Wittmann et al. [4] found that the resulting interpolations in Figure 1A-C are either not smooth or do not adequately reproduce the Boolean functions they should interpolate. In response, they introduced the minimal degree polynomial BooleCube interpolation which is smooth and reproduces the Boolean function for all input vectors in {0,1}^*n*^.

For a Boolean function *B* : {0, 1}^*n*^ → {0, 1}, the BooleCube interpolation *C*[*B*(*x*_1_,…,*x*_*n*_)] is given by

$$\begin{array}{c} C\left[B\left(x_{1},\ldots ,x_{n}\right)\right]=\overset{1}{\underset{{\bar{x}}_{1}=0}{\sum }}\overset{1}{\underset{{\bar{x}}_{2}=0}{\sum }}\ldots \overset{1}{\underset{{\bar{x}}_{n}=0}{\sum }}\\ \;\times \left[B\left({\bar{x}}_{1},\ldots ,{\bar{x}}_{n}\right)\overset{n}{\underset{i=1}{\prod }}\left(x_{i}{\bar{x}}_{i}+\left(1-x_{i}\right)\left(1-{\bar{x}}_{i}\right)\right)\right] \end{array}$$

As an example consider the Boolean function *B* ' (*a*, *b*) = *a* OR *b*. The BooleCube interpolation is *C*[*B* '] = *a* + *b* - *ab* which satisfies *C*[*B* ' (*a*, *b*)] = *B* ' (*a*, *b*) for all (*a*,*b*) ϵ {0,1}^2^ and is smooth (Figure 1D).

Wittmann et al. [4] also extended this formalism to include a switch-like behavior of network nodes by modifying the inputs to the BooleCube interpolation by a sigmoid-shaped Hill function *f*(*x*) = *x*^*n*^ / (*x*^*n*^ + *k*^*n*^) leading to HillCubes and, with a normalized sigmoid function *f*(*x*) = (*x*^*n*^/(*x*^*n*^ + *k*^*n*^))/(1/(1 + *k*^*n*^)), to normalized HillCubes (Figure 1E).

These high degree polynomial interpolations of Boolean functions are implemented in the Matlab package Odefy [6]. While the SQUAD model can be simulated efficiently even for complex network topologies, Odefy’s implementation of the polynomial interpolation exhibits a time complexity in Ω(2^*n*^) (where *n* is the number of inputs to the node with the most inputs) for the creation of the model as well as for its simulation.

In extension of such approaches we show how a tree data structure to store the functions of the network leads to a straightforward and efficient way to calculate the polynomial interpolation for almost any example of practical importance, thereby greatly expanding the range of networks that can be simulated and analyzed in reasonable time using this model. Since semi-quantitative models allow for a range of new analysis techniques such as sensitively quantifying the basins of attraction of the stable states or the influence of noise on network behavior, this paves the way for additional insights into network dynamics. We demonstrate this new algorithm as a part of Jimena, a new Java GRN simulation framework which focuses on computational efficiency and a modularized architecture to facilitate the development and testing of new algorithms and models surrounding GRNs.

## Implementation

### A recursive algorithm to calculate the BooleCube polynomial

**Figure 2:**
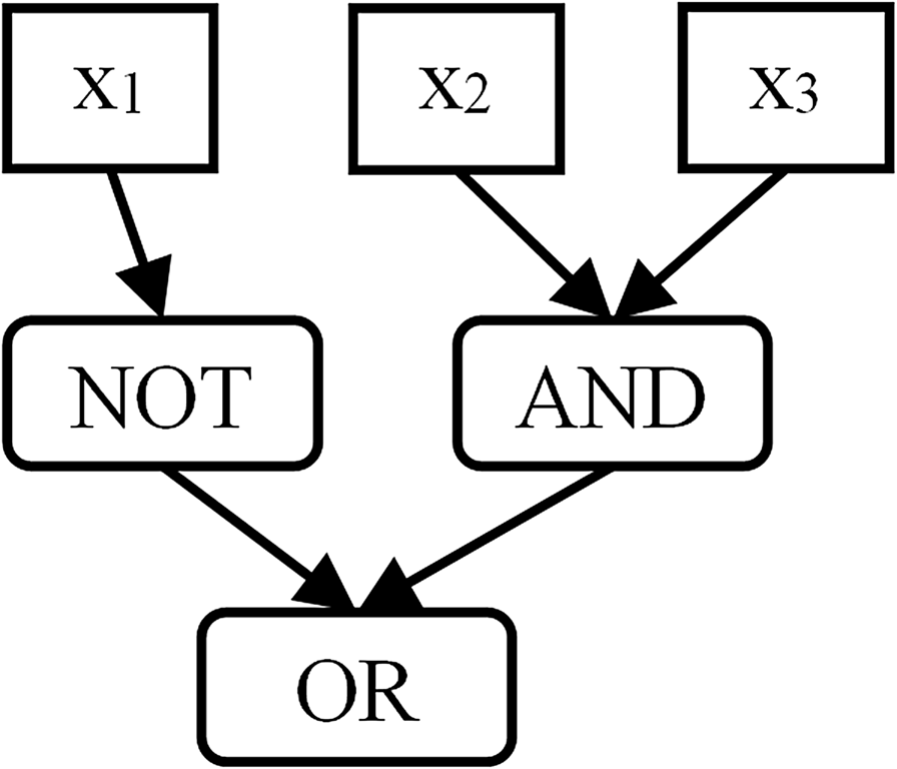
**A Boolean tree for the function *B***_**1**_***(x***_**1**_**, *x***_**2**_**, *x***_**3**_**) = (*NOT x***_**1**_**) *OR (x***_**2**_** AND *x***_**3**_**).** Input variables *x*_*i*_ are connected by Boolean operators. The OR node is the root of the tree, i.e. its value determines the value of the function represented by the tree.

To tackle the space and time complexity issues of the polynomial interpolation present in previous implementations, we use simple Boolean trees to represent the Boolean functions of the network. In a Boolean tree (Figure 2), the leaves (i.e. nodes without ingoing connections such as *x*_1_ in Figure 2) are inputs to the function, and the non-leaf nodes are unary or binary Boolean gates (such as AND in Figure 2). Each Boolean gate combines the values from its ingoing connections to an outgoing line in accordance with the Boolean function (e.g. AND, OR or NOT) the gate represents. The value of the root node, i.e. the unique node without outgoing connections to other nodes, determines the value of the function.

Boolean trees can be straightforwardly created in linear time by parsing the Boolean expression which defines a Boolean function, and the function represented by the tree can be interpolated very quickly using a recursive algorithm which we will describe in detail below.

While Odefy, which uses exhaustive value tables stored as multidimensional arrays to represent the functions, needs a space and time in Θ(2^*N*^) to store a function where *N* is its arity, a Boolean tree guarantees a space requirement in O(*E*) where *E* is the length (in characters) of the Boolean expression used to input the desired Boolean function. Jimena’s time complexity can therefore be minimized by minimizing the description length of the Boolean functions or by minimizing the Boolean trees, respectively. This difference in time complexity between Odefy and Jimena is of high practical importance, since Boolean functions that appear in today’s GRNs can almost exclusively be described by Boolean expressions of moderate length. For most GRN design patterns (e.g. activator-inhibitor-patterns) we even get functions for which their expression length *E* is in O(*N*). A plethora of published networks are constructed using these patterns. These may feature nodes with more than ten inputs per node, for example the floral organ development network presented in [7], the automatically generated regulatory network for arthritis therapy responders from [8] or the plant immunity network from [9] which we will analyze below.

In addition to the speed up of the creation of the GRN, the tree structure also expedites the calculation of BooleCube (and therefore HillCube) interpolations since we can essentially apply the interpolation separately to all logic gates of the function and recursively evaluate the tree from the root node to the leaves. For a more precise description of the algorithm consider a regulatory network with nodes {*x*_1_,…,*x*_*n*_}. May the Boolean function *B*_*k*_(…) of a node *x*_*k*_ be given by a Boolean tree consisting of nodes {*n*_1_,…,*n*_*m*_}. Note, that as shown in Figure 2, these *n*_*i*_ represent binary or unary Boolean gates or inputs to the function *B*_*k*_(…). For each function in the network we get a separate tree and therefore a separate set {*n*_1_,…,*n*_*m*_} .

To illustrate the relationship between {*x*_1_,…,*x*_*n*_} and {*n*_1_,…,*n*_*m*_} consider the network {*x*_1_,*x*_*2*_} where *B*_1_(*x*_1_,*x*_2_) = *x*_1_ AND *x*_2_ and *B*_2_(*x*_1_,*x*_2_) = *x*_1_ OR *x*_2_. A possible Boolean tree for the function *B*_1_ could then be given by the nodes *n*_1_,*n*_2_,*n*_3_, where the root node *n*_1_ is an AND node with the leaves *n*_2_ and *n*_3_, *n*_2_ is an input node representing *x*_1_ and *n*_3_ is an input node representing *x*_2_.

We call the function given by the subtree whose root is *n*_*i*_*f*_*i*_, where *f*_*i*_(*x*_*j*_) = *x*_*j*_ for some *x*_*j*_ for all input nodes. If a node *n*_*i*_ is not an input node to the network we call its binary or unary logic gate ⊗ _*i*_. In our example Boolean tree from above we would get *f*_2_(*x*_1_) = *x*_1_, *f*_3_(*x*_2_) = *x*_2_, *f*_1_(*x*_1_) = *x*_2_ = *x*_1_ AND *x*_2_ and ⊗ _1_ = AND.

For an arbitrary Boolean function *f* : {0, 1}^*τ*^ → {0, 1}, *C*[*f*] denotes its BooleCube interpolation. We can then construct a recursive term for the interpolation *C*[*f*_*i*_] of a node *n*_*i*_ ’s function *f*_*i*_ using the following rules:

If *n*_*i*_ represents an input node of the tree for which *f*_*i*_(*x*_*j*_) = *x*_*j*_ we set *C*[*f*_*i*_] ≡ *x*_*j*_

If *n*_*i*_ is a unary negating node whose input is a node *n*_*j*_, we set

$$C\left[f_{i}\right]=C\left[{⊗}_{i}\left(f_{j}\right)\right]\equiv C\left[{⊗}_{i}\right]\left(f_{j}\right)=C\left[\neg \right]\left(f_{j}\right)=1-C\left[f_{j}\right]$$

If *n*_*i*_ is a binary node with two inputs *n*_*j*1_ and *n*_*j*2_ whose functions are *f*_*j*1_ and *f*_*j*2_ we set

$$\begin{array}{c} C\left[f_{i}\right]=C\left[f_{j_{1}}⊗f_{j_{2}}\right]\equiv C\left[⊗\right]\left(C\left[f_{j_{1}}\right],C\left[f_{j_{2}}\right]\right)\\ \;=\overset{1}{\underset{{\bar{a}}_{1}=0}{\sum }}\overset{1}{\underset{{\bar{a}}_{2}=0}{\sum }}\left({\bar{a}}_{1}⊗{\bar{a}}_{2}\right)\cdot \overset{2}{\underset{\varphi =1}{\prod }}\xi \left(C\left[f_{j_{\varphi }}\right],{\bar{a}}_{\varphi }\right)\; \end{array}$$

where ξ(*α,β*) is an abbreviation for *αβ* + (1-*α*)(1-*β*). Notice that this term collapses to *f*_*j*1_*f*_*j*2_ if ⊗ _*i*_ = ∧ (i.e. the logic gate is an AND) and $f_{j_{1}}+f_{j_{2}}-f_{j_{1}}\cdot f_{j_{2}}$ if ⊗ _*i*_ = ∧ (i.e. the logic gate is an OR), both of which can be calculated very efficiently. The *C*[…] parts of the terms above are then evaluated using the same rules until all branches of the recursion have reached an input node.

If we apply this algorithm to the root node of the network we get the interpolation *C*[*B*_*k*_] of the function *B*_*k*_. An overview of the algorithm written in pseudo code as well as a proof that the result of this algorithm is identical to the high degree polynomial defined in [6] can be found in Additional file 1.

For our example network we get *C*[*f*_1_(*x*_1_, *x*_2_)] = *C*[*AND*](*f*_2_(*x*_1_), *f*_3_(*x*_2_)) = *f*_2_(*x*_1_) ⋅ *f*_3_(*x*_2_) = *x*_1_ ⋅ *x*_2_. As a second example consider the function *B*_1_ (Figure 2). Traversing the tree starting from the root node *n*_*OR*_ we get *C*[*B*_1_] = *C*[*f*_*OR*_] = *C*[*f*_*NOT*_] + *C*[*f*_*AND*_] - *C*[*f*_*NOT*_]*C*[*f*_*AND*_] = (1 - *x*_1_) + *x*_2_*x*_3_ - (1 - *x*_1_)*x*_2_*x*_3_.

### Obtaining the stable steady states for discrete models from the Boolean tree

As a side effect, Boolean tree data structures instead of value tables also expedite and simplify the creation of binary decision diagrams (BDDs) equivalent to the Boolean functions of the network (see [10] for a comprehensive review of BDDs and their algorithms).

**Figure 3:**
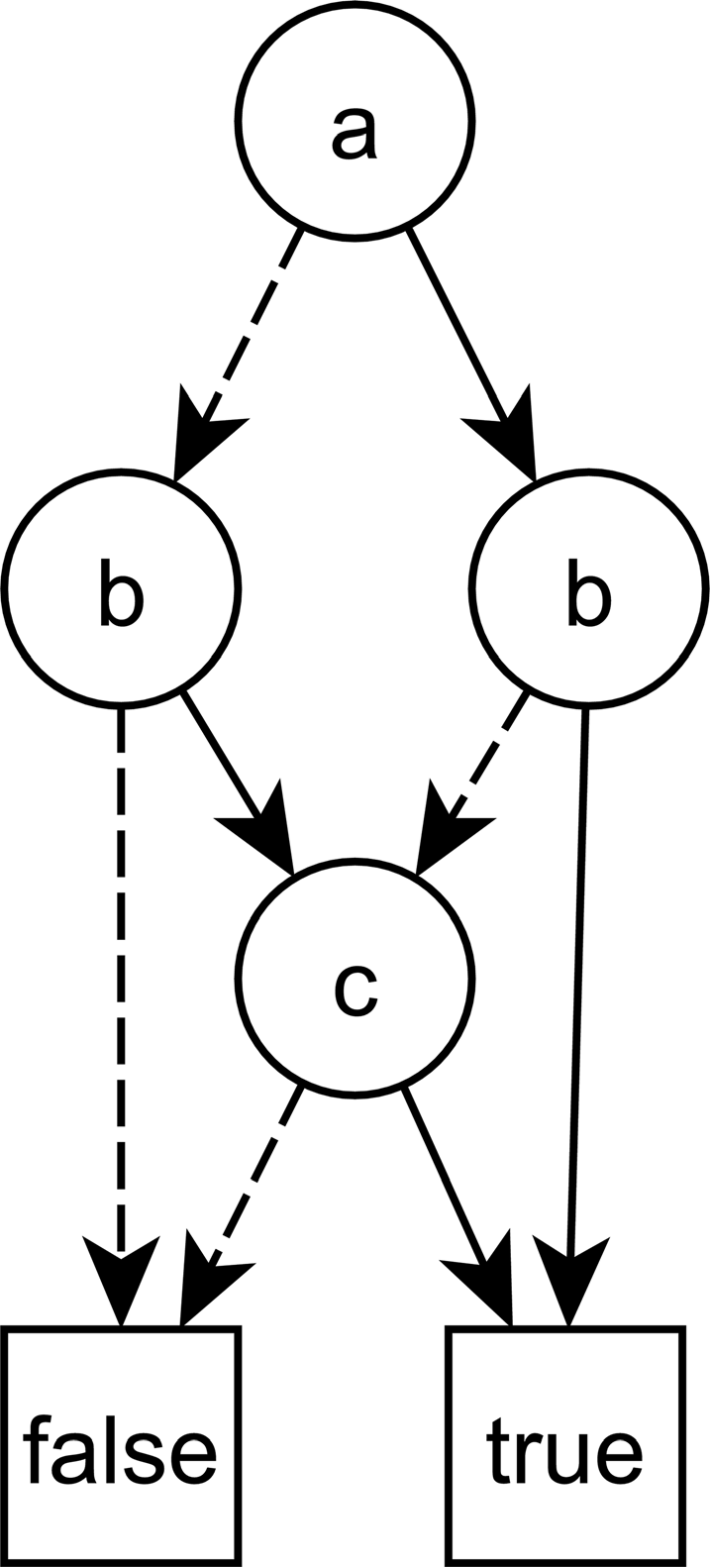
**A binary decision diagram for the function (a OR b) AND (b OR c) AND (a OR c).** Evaluation starts at the node “**a**”, which does ***not*** feature any ingoing connections from other nodes. If the value of the node is 1, the solid line is followed, if it is 0, the dashed line is followed. For the input values a = c = 1 and b = 0 one would go down from the “**a**” node to the right “**b**” node, on to the “**c**” node and finally along the solid line to the “true” node. This corresponds to (1 OR 0) AND (0 OR 1) AND (1 OR 1) = 1 AND 1 AND 1 = 1 (true).

BDDs, whose algorithmic potential was first investigated by Bryant et al. [11], represent Boolean functions in a rooted, directed and acyclic graph. The structure and the evaluation of BDDs is explained in Figure 3. Common problems surrounding Boolean functions such as finding all solutions satisfying a given expression can efficiently solved once a BDD representation has been created using a set of standard algorithms [11], which is not possible with Boolean trees.

A possible application of BDDs is the search for all stable steady states (SSS) in discrete models, i.e. network states which reproduce themselves in each following step of a discrete simulations. In contrast, a temporary state will be left if the system is simulated. The calculated steady states can be enumerated and applied in systems biology (e.g. [9], reviewed in [12]). Furthermore, BDDs based search algorithms and random sampling algorithms to approximate the stable states in continuous networks have been explored ([3,2]).

If *B*_*i*_ are the Boolean functions defining a network consisting of the nodes *x*_*i*_, a network state *x*_*i*_*,*…,*x*_*n*_ is a stable steady state [3] by definition if and only if $\;\Lambda_{i}\left(B_{i}\left(x_{i,1},\ldots ,x_{i,n_{i}}\right)=x_{i}\right)=\mathrm{true}$ where *x*_*i,j*_*∈*{*x*_*i*_*,*…,*x*_*n*_} is the *j*-th input to the function *B*_*i*_ . In other words, all Boolean functions must evaluate to the value which their target node already holds. In common BDD frameworks, such as the JavaBDD framework [13] which is used by Jimena, BDDs can be constructed by combining elementary BDDs (e.g. BDDs equivalent to the Boolean function consisting of the value of a single input variable) using logical operators such as AND or NOT. For example, to construct a BDD for the function *B* ' (*a*, *b*) = *a* OR *b* one would create the BDDs *BDD*(*a*) and *BDD*(*b*) for the inputs *a* and *b*, and use the framework to contruct *BDD*_*OR*_(*BDD*(*a*),*BDD*(*b*)) where *BDD*_*OR*_ constructs the logical OR of two BDDs.

Recursively traversing the Boolean tree of the functions *B*_*i*_, the BDDs of these functions can be straightforwardly constructed in the framework by the synthesis method described above, and then combined to a BDD for the expression $\;\Lambda_{i}\left(B_{i}\left(x_{i,1},\ldots ,x_{i,n_{i}}\right)=x_{i}\right)=\text{true}$. All satisfying assignments for this equation, which are identical to the stable steady states of the network, can then be found by standard algorithms in the BDD framework.

In essence, Boolean trees are necessary to speed up the simulation of continuous networks, while BDDs are essentials for the efficient calculation of SSS.

## Results and discussion

A jar-library version of Jimena, its sources code, a ready-to-use Eclipse workspace including a commented usage example, further documentation and example networks are available [14].

### Speed up of the BooleCube calculation

While it takes a time in Θ(*N*⋅2^*N*^) to compute previous implementations of the polynomial interpolation, the tree algorithm runs in a time in O(*E*) (where *E* is the description length of the Boolean function) since the (at most second degree) polynomials of each node can be evaluated in O(1) and the number of nodes is in O(*E*). This also implies that a GRN given by a set of Boolean expressions can be interpolated, and therefore simulated, in a time proportional to the description length of the network.

To benchmark the time needed to simulate a network with a given node degree we used a scalable artificial network topology which features 2.5*n*-5 interactions and a maximum degree of *n* for *n* nodes (see Additional file 1 for a detailed definition of the network). The simulation was run for 10 simulation-time seconds and the normalized HillCube mode [6] was used in Jimena and Odefy.

Directly comparing the simulation speed of Odefy and Jimena is not trivial since the time needed by Odefy to simulate a network for a given time t does not depend on this parameter, since the simulation accuracy decreases with higher time t simulated.

**Figure 4:**
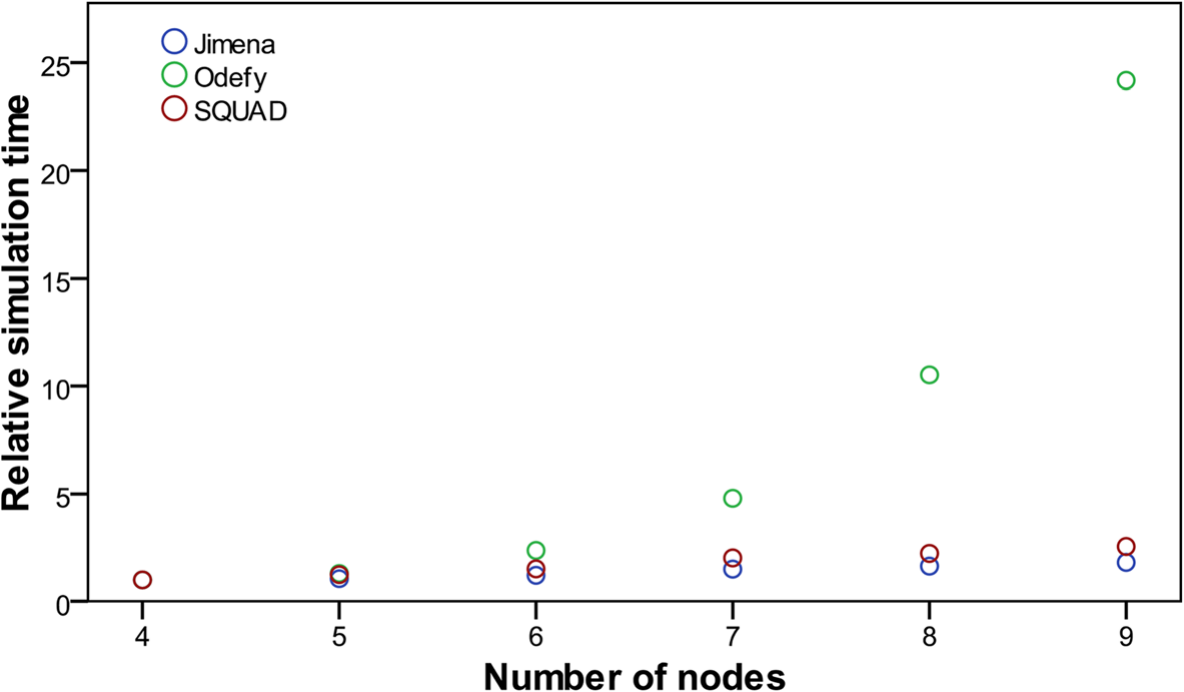
**Simulation time for a continuous model.** x-axis: number of involved nodes. y-axis: Time (in seconds) to simulate a standardized network with the given number of nodes. Note that in all figures the number of nodes refers to the number of actual network nodes x_i_ as opposed to the number of nodes in the Boolean tree. To highlight the time complexity of the different calculation methods, the data series are scaled to coincide for a network with 4 nodes. Actual simulation times for 4 nodes: Jimena (red) = 0.019 s, Odefy (blue) = 0.040 s, Squad (green) = 0.046 s. The Additional file 1 explains the definition of the network for a given number of nodes.

Jimena, on the other hand, uses a standard fixed-step forth-order Runge–Kutta method to simulate the networks, hence its performance greatly depends on the step size of this solving method. For Figure 4 we therefore scaled the time needed by Jimena such that the data series coincide for networks with 4 nodes. Without the scaling Jimena takes about 20 ms for the simulation with a step size of 1 ms. The experimental data confirm the exponential increase in calculation time for Odefy and a linear one for Jimena. A separate benchmark for the model creation time yielded similar results.

Since we chose test networks for which analogous activator-inhibitor-networks could be constructed, we were also able to benchmark the simulation of the equivalent networks using the octave code obtained from the SQUAD Export-to-Octave function. As one would expect from the design of the differential equations, the integration of the ODEs from the SQUAD model exhibits a linear time complexity with respect to the maximum degree of the network nodes.

While this example shows Jimenas performance for high node degrees, it does not cover networks with large numbers of nodes. We therefore compared the runtime behavior of BooleCube interpolations in Odefy and Jimena in small to large size networks created by the random Erdős–Rényi paradigm, where a connection between nodes are set with equal probability, and by the random scale-free paradigm, where the node degree distributions follows a power law, i.e. the number of network nodes with *k* connections to other nodes is proportional to *k*^-λ^ where λ is a constant usually between 2 and 3. It has been established that a large majority of naturally occurring networks are scale-free (see [15] for a review).

**Figure 5:**
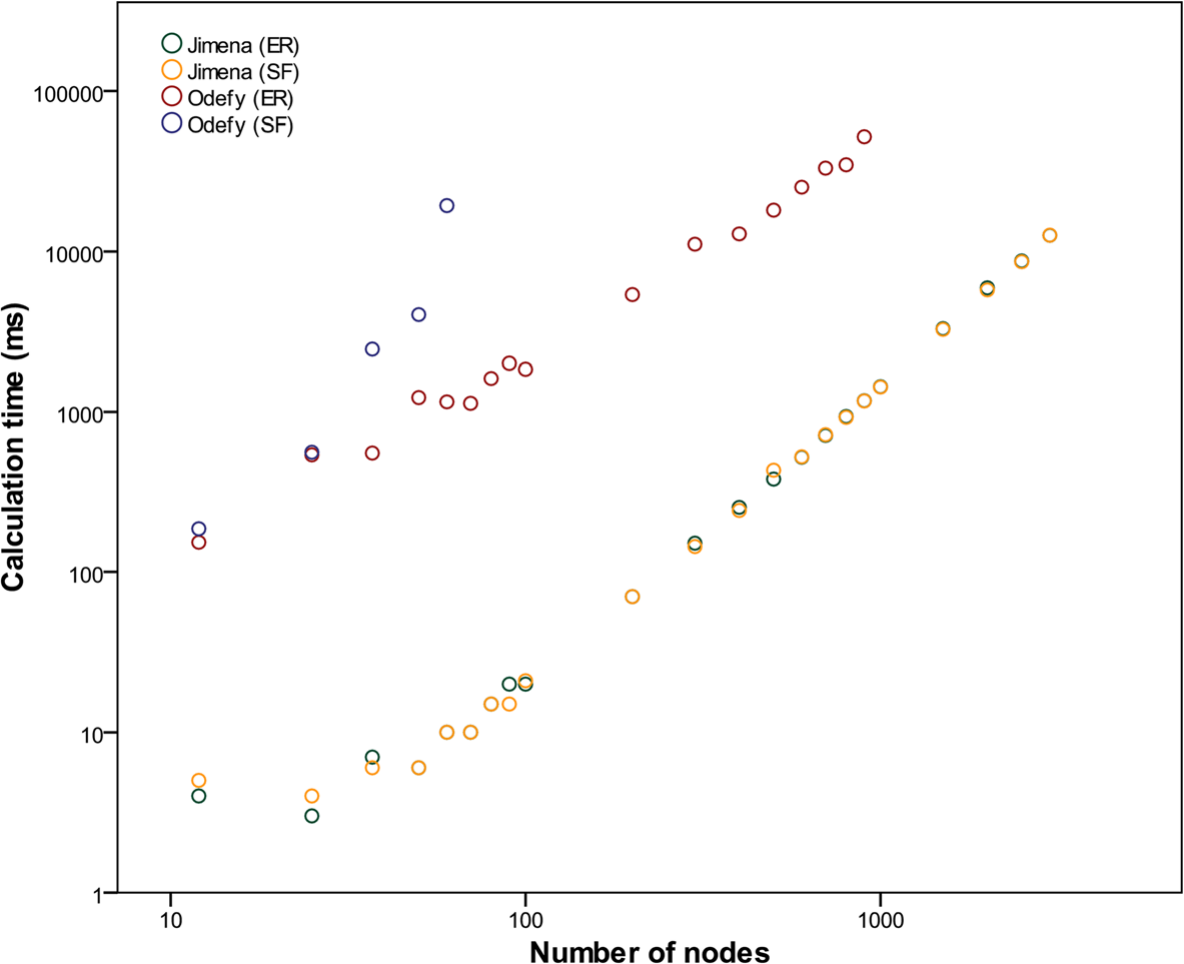
**BooleCube network performance of Jimena and Odefy.** Random Erdős–Rényi and scale-free networks with a given number of nodes **n** and 3·n interactions (*arrows*) were simulated for 10 seconds with a step size of 0.05 s in Jimena. The scale-free networks were grown using a preferential attachment mechanism. All simulations were aborted after a maximum of 1 minute calculation time.

The run times (creation and simulation) are plotted in Figure 5. Note how Odefy performs especially unfavorably for scale-free networks, which by definition tend to contain nodes with very high degrees, while Jimena’s run time reacts benignly to increases in node degree, number of connections and number of nodes. The simulation of scale-free networks with 70 nodes or more did not terminate in Odefy even after runtimes of several hours. Additional benchmarking of large HillCube and normalized HillCube networks yielded almost identical results.

Since SQUAD, BooleanNet and other simulation frameworks cannot simulate BooleCube networks, they are not included in this comparison. With the limitation to networks consisting only of simple activating or inhibiting influences, SQUADs runtime behavior is similar to that of Jimena (cf. Figure 4).

### Speed of the SSS calculation

**Figure 6:**
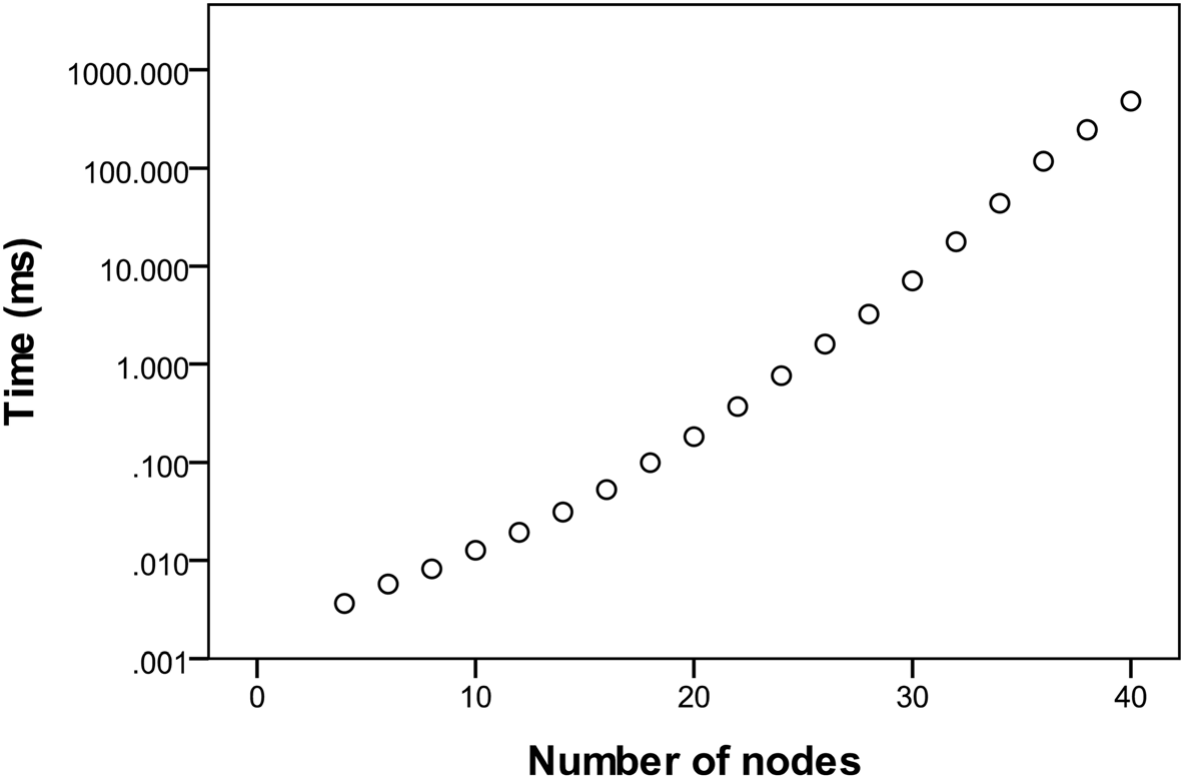
**Stable state calculation time for a discrete model.** x-axis: number of involved nodes. y-axis: Time (in milliseconds) needed to determine the stable states of a standardized network with the given number of nodes. The Additional file 1 explains the definition of the network for a given number of nodes.

Since the number of SSS in discrete models can be in Θ(2^*E*^) this is also the minimum time complexity of a search algorithm. To benchmark our implementation we used the same scalable test topology as before which features 2^(*n*-2)/2^ SSS for *n* nodes. In Figure 6 one can see that the time needed to determine the SSS stays very slow even for high node degrees. It only begins to increase for high numbers of solutions (40 nodes: 524,288 solutions) where it grows linearly with the number of solutions. Note that the exponential increase of the number of SSS with the size of the network is a artificial worst case scenario and that for evolutionary reasons, many GRN only feature a limited number of stable steady states.

For medium sized random scale-free networks (100 nodes, 200 interactions, 100 unique networks) we obtained a mean run time of 3890 ms (median 1401 ms). Further experimentation showed that the calculation of the stable steady states using JavaBDD as a BDD framework is usually possible for random networks until about 150-200 network nodes and 500 interactions on standard hardware, with the limit being the main memory available in the computational environment.

Since larger networks for which Jimena takes a measurable time to calculate the SSS cannot be loaded in Odefy, we could not directly compare the two frameworks in this respect.

This time complexity makes the search feasible even for larger and highly interconnected networks which could not even be loaded using a multidimensional array implementation.

### Multithreading

To determine as many stable steady states of a network as possible for continuous models such as the Odefy and SQUAD models, it is necessary to exhaustively sample a large state space. This task can be greatly expedited by distributing the sampling to multiple CPU cores as done automatically by the search algorithms implemented in Jimena.

Since Jimena’s tree-based networks are very lightweight compared to multidimensional array implementations, they can be copied quickly and many of them can be held in memory at the same time. This not only allows for an excellent scaling behavior on commonly used multi-core systems, resulting for example in an almost 8 times higher sampling rate on an 8 core system, but also facilitates the efficient comparison of variants of a given network to analyze its stability with regard to certain manipulations such as null mutations [7]. The usage of the stable state searching algorithm is explained the aforementioned commented usage example. As applied examples we studied network behavior in plants and bacteria.

### Applied example: Arabidopsis thaliana development

The first example takes the plant *Arabidopsis thaliana* flower organ specification network from [7]. It concerns A. thaliania development and makes use of Boolean functions instead of mere activating and inhibiting influences and features nodes with a larger number of inputs. Floral homeotic protein APETALA 1 is a central transcription factor in this network. It promotes early floral meristem identity in synergy with LEAFY and regulates positively the B class homeotic proteins APETALA3 and PISTILLATA with the cooperation of LEAFY and its co-regulator UFO (unusual floral organs). The network we investigate here [7] is a discrete model on the ABC homeotic floral genes, summarizing also non-ABC gene interactions to a dynamical floral organ cell fate model.

We simulated the network for 10 simulation-time seconds using the normalized HillCube (NHC) model on a standard 2.67 GHz CPU. For the step size of Jimena’s ODE solver we tested 0.01 s and 0.1 s. Even with a step size of 0.1 s, the absolute error of the simulation is in the order of 10^-5^ when simulating 10 seconds and much lower when searching for a stable state, which should already be more than enough for practical applications. To benchmark the original SQUAD ODEs we used the simplified activator-inhibitor version of the network shown in Figure 1 from [7], although we are aware that in the same article the authors propose a slightly modified version of the standardized qualitative dynamical systems methodology implemented in SQUAD. 10 successive simulations of the network for Jimena, SQUAD and Odefy showed that Odefy needs 467(±2) ms to create the network and additional 583(±4) ms to simulate it, while the SQUAD ODEs are solved in 467(±0.3) ms. With step sizes of 0.1 s and 0.01 s Jimena needs for these two tasks only 3.1(±0.4) ms and 28.2(±0.7) ms respectively (Table 1). We used this computational improvement to determine the basins of attraction of the stable states of the network assuming a NHC model based on the Boolean function given in the corrigendum to [7], a calculation which also benefits greatly from Jimenas automatic multithreading. For the co-regulator UFO we assumed a loop (UFO = UFO) which reproduced the known stable states in Jimenas discrete und NHC model calculations. By simulating the network from 10^6^ random initial states we found that although both models are based on the same Boolean functions, interestingly the inflorescence states INF1, INF2, INF3 and INF4 (inflorescence attractors 1-4), whose biological validity has been confirmed by gene expression experiments [16], are much more unstable in the NHC model, having a combined basin of attraction size of only 0.06% as opposed to 5.1% in the discrete model and 17.6% in the continuous model from [7] (Table 2). In other words, when simulated from 10^6^ initial states where the values of the nodes have been chosen randomly from the interval [0,1] only 0.06% of the simulations converge on a state corresponding to a non-flowering phenotype.

**Table 1:** Loading and simulation time in different simulation frameworks

**Package**	**Calculation**	**Time (ms)**
Odefy	Network loading	467(±2)
Odefy	Network simulation	583(±4)
SQUAD	Network simulation	467(±0.3)
Jimena	Network loading	3.7(±0.2)
Jimena	Network simulation (time step: 0.1 s)	3.1(±0.4)
Jimena	Network simulation (time step: 0.01 s)	28.2(±0.7)

The *Arabidopsis thaliana* flower organ specification network was loaded and simulated ten times in Odefy, SQUAD and Jimena, the mean calculation time is given in the table.

**Table 2:** Basins of attraction of the A. thaliana development network

**Attractor**	**NHC model (%)**	**Discrete model (%)**	**Continuous model (%)**
INF1	0.005	1.66	4.74
INF2	0.016	1.66	4.77
INF3	0.010	0.88	4.01
INF4	0.032	0.88	4.06
SEP	0.144	9.91	11.01
PET1	0.477	10.05	12.74
PET2	0.024	0.14	1.89
STM1	74.556	37.4	28.46
STM2	7.920	1.15	6.54
CAR	16.816	36.25	21.79

An NHC model based on the Boolean functions of the corrigendum to [7] was simulated from 10^6^ random initial vectors. The parameters of the Hill function were n = 2 and k = 0.5 and the decay parameter was τ = 1 for all nodes. The values for the corresponding discrete and continuous model from the original article are cited from there [7].

Using active EMF1 (embryonic flower 1) and TFL1 (terminal flower 1) nodes (i.e. EMF1 > 0.5 AND TFL1 > 0.5) as an indicator of an inflorescence state, we then determined the basins of attraction of the same model assuming null mutations for all 42 interactions (arrows) of the network by simulating from 10^4^ random start vectors per mutant. The combined basin of attraction size of each mutant stayed below 0.5%, except for a removal of the influence of AP1 (APETALA1) on TFL1 whose mutation directly causes our condition for inflorescence state to fail, leading to a combined basin size of ~3.5%.

These results corroborate the hypothesis that the inflorescence attractors are transitory in nature, such that small perturbations lead to progress in plant development and cell differentiation arriving at few and robust standard outcomes of floral organs. Furthermore, the low size of the inflorescence basins of attraction of the mutant networks is consistent with a reported strong robustness of *A. thaliana* mutants against a non-flowering phenotype [17].

### Applied example II: Arabidopsis thaliana immunity and pathogen Pst DC3000

A second example considers a different area, the immune response of the *Arabidopsis thaliana* plant against gram negative bacterium *Pseudomonas syringae DC3000 pv tomato* and its modulation by cytokines [9]. Furthermore, this interaction network concerns two organisms, plant and its pathogen and is already due to this fact more complex. The larger Boolean network features 104 nodes and 156 interactions. In particular, using the immune response marker node PR1 the counteracting or synergistic effects of different hormone and cytokine can be modeled. For instance, cytokine enhances immune responses while auxin stimulates growth but mitigates immune defence. A number of further insights were obtained from this network and its analysis including new cytokine mediated regulatory interactions and specific synergism between cytokinin and salicylic acid pathways as well as differences in network responses for fully virulent and mitigated pathogens [9]. With a step size of 0.1 s and 0.01 s Jimena needs 20.0(±0.4) ms and 193(±3) ms respectively to simulate 10 seconds using the BooleCube model, while SQUAD takes 11.04(±0.02) s. In Odefy the network cannot be loaded due to the high number of inputs to some nodes. Since in Jimena null mutations are part of the computational core, networks can be mutated and restored very quickly. This not only includes the removal of nodes, but also the removal of single interactions between network nodes. We searched for the stable states of the network for all single null mutations (n = 156) in the continuous NHC model by randomly sampling the state space with 2000 initial states per mutation, and compared the resulting stable states with the ones obtained by treating the network as a discrete Boolean model. We found that for all mutants and the original network the stable states of the discrete and the NHC model seem to be identical.

As we expected, the network exhibits a strong robustness against null mutations, with only 2 mutations changing the number of stable states (from 2 to 1). These are the null mutation of the influence of SA (salicylic acid) on ROS (reactive oxygen species) and of ROS on SA where SA is a key hub node of the network and the small cycle SA → RO → SA is crucial for its number of stable states. For all other mutations (n = 154) the changes of the stable states are minor, with only one mutation effecting more than four changed nodes per stable state, namely the removal of ETR/CTR1 (ethylene response / cytosolic serine/threonine kinase constitutive triple response 1) → AHP (Histidine-containing phosphotransmitters) which causes five nodes to change, and most single mutations (n = 142) leading to no change at all.

To check whether the number of stable states increases assuming multiple mutations we then determined the stable states for up to 4 null mutations (n ≈ 2.4⋅10^7^) in the discrete network model and found that the number of stable states never exceeds 2. Using a single 2.67 GHz CPU core Jimena constructs and analyzes 2,700 mutants per second in this network, and more than 24,000 mutants per second in the *A. thaliana* development network from above (number of interactions = 42), which demonstrates its computational efficiency even for complex networks. The result of this analysis hints to the robustness of the network against the emergence of new stable states even when multiple interactions (up to four) are removed. Inbuilt robustness is not a rare phenomenon in biological signaling cascades as independently confirmed from experimental data such as promoter recombination trials in *E. coli*[18] or the reported phenotypical robustness of *C. albicans* against null mutations of transcriptional regulators [19]. Additional benefits from robustness for this particular signal cascade include that additional stable states could be detrimental to the latency and efficacy of immune reactions.

## Conclusion

Within the last years the size and complexity of discovered genetic regulatory networks has increased substantially, partly due to automated network creation techniques using time series data from methods such as real-time RT-PCR or RNAseq.

Motivated by current limitations of Odefy (version 1.18, year 2013), the use of tree data structures and corresponding algorithms in Jimena paves the way for the simulation and analysis of more sophisticated networks than possible previously, including those beyond the scope of simple activating and inhibiting influences covered by SQUAD. This may provide additional insight especially with regard to the role of nodes that are influenced by many other nodes, which seem to greatly influence the behavior of many GRNs.

**Figure 7:**
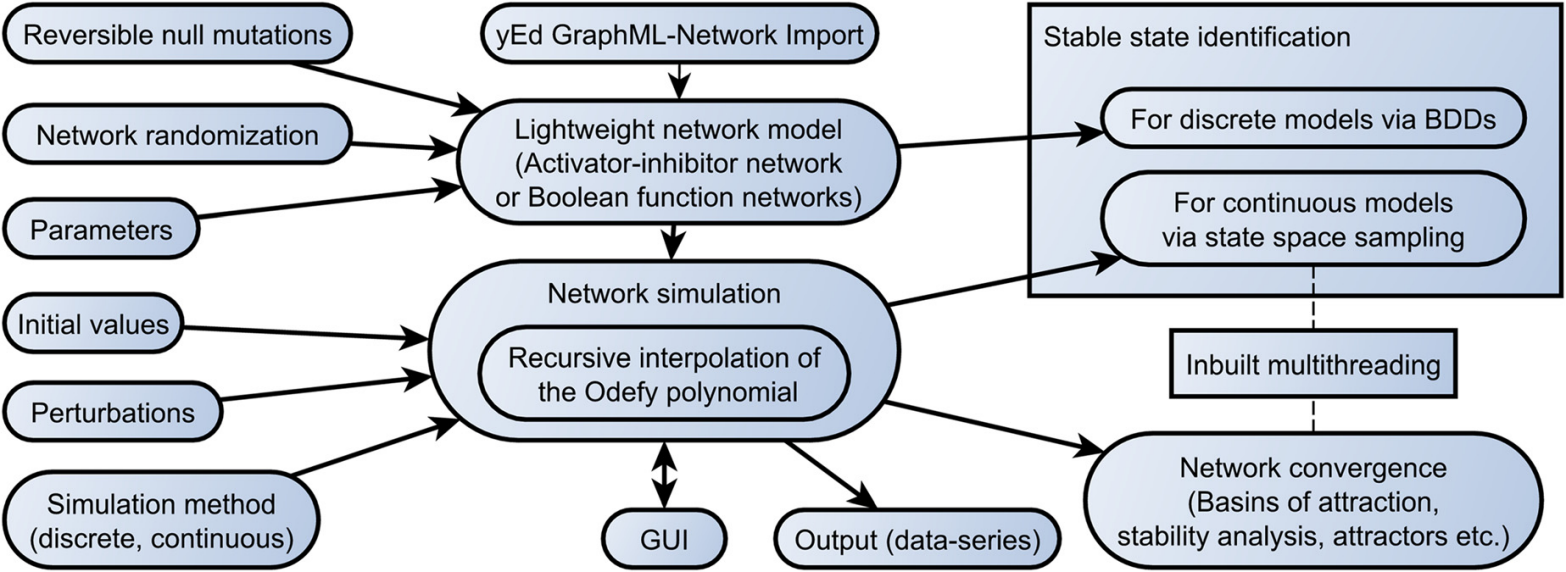
**Feature overview of the Jimena simulation framework.** Included are all features as of version 160913.

For an overview of all currently published features of Jimena see Figure 7.

## Availability and requirements

The software, its source code, example data and a tutorial are available from http://stefan-karl.de/jimena/ and http://www.bioinfo.biozentrum.uni-wuerzburg.de/computing/jimena. Jimena runs on any operating system (windows, Linux, Mac). Jimena requires Java 7 or above.

## Competing interests

The authors declare that they have no competing interests.

## Authors’ contributions

SK developed, formally verified, implemented and benchmarked the algorithms, contributed ideas and algorithms to the applied examples and wrote the first draft of the manuscript. TD conceptualized and analyzed the applied examples and the biological insights, reviewed and revised the manuscript and led the project. Both authors have read the manuscript and approved the final version.

## Supplementary Material

Additional file 1 In this document file (.doc) we include a proof summary of the BooleCube interpolation algorithm, the topologies for the benchmarks used and the pseudocode for the interpolation algorithm. Click here for file
