# Supplementary material for: Jimena: efficient computing and system state identification for genetic regulatory networks
Source: BMC Bioinformatics. 2013 Oct 11;14:306. doi: 10.1186/1471-2105-14-306 (PMC3853020; doi:10.1186/1471-2105-14-306)
Supplement: Additional file 1 — In this document file (.doc) we include a proof summary of the BooleCube interpolation algorithm, the topologies for the benchmarks used and the pseudocode for the interpolation algorithm. [file 1471-2105-14-306-S1.docx]

# Additional File 1

## Proof summary of the BooleCube interpolation algorithm

We prove that the interpolation function $C[B]$ of a Boolean interaction function $B(...)$ with tree nodes $n_{i}$ in a network with nodes $x_{i}$ can be assembled stepwise from the leaves (= inputs) of the tree using the algorithm from the Algorithms section by first considering input nodes, i.e. nodes $n_{k}$ for which $f_{k}(x_{j})=x_{j}$ for some$x_{j}$. We can determine $C[f_{k}]$ by simply applying the Odefy polynomial:

$$C[f_{k}(x_{j})]=\sum_{\bar{x}_{j}=0}^{1} f_{k}(\bar{x}_{j})\prod_{i=j}^{j} \xi\left(x_{i},\bar{x}_{i}\right)=\sum_{\bar{x}_{j}=0}^{1} f_{k}(\bar{x}_{j})\xi\left(x_{j},\bar{x}_{j}\right)=f_{k}(x_{j})=x_{j}$$

The second case we distinguish in the algorithm are negating nodes, the only unary nodes in the tree. For a negating node $n_{k}$ whose input node $n_{i}$ has a function $f_{i}(x_{1},...,x_{n})$ we show that $C[f_{k}]=C[\neg f_{i}]=1-C[f_{i}]$:

$$1-C\left[f_{i}\right]=1-\sum_{\bar{x}_{1}=0}^{1} \cdot...\cdot\sum_{\bar{x}_{n}=0}^{1} f_{i}\left(\bar{x}_{1},...,\bar{x}_{n}\right)\cdot\prod_{i=1}^{n} \xi\left(x_{i},\bar{x}_{i}\right)$$

By setting

$$1=\sum_{\bar{x}_{1}=0}^{1} \cdot...\cdot\sum_{\bar{x}_{n}=0}^{1} 1\cdot\prod_{i=1}^{n} \xi\left(x_{i},\bar{x}_{i}\right)$$

which can be easily proven by induction over $n$we get

$$=\sum_{\bar{x}_{1}=0}^{1} \cdot...\cdot\sum_{\bar{x}_{n}=0}^{1} 1\cdot\prod_{i=1}^{n} \xi\left(x_{i},\bar{x}_{i}\right)-\sum_{\bar{x}_{1}=0}^{1} \cdot...\cdot\sum_{\bar{x}_{n}=0}^{1} f_{i}\left(\bar{x}_{1},\ldots,\bar{x}_{n}\right)\cdot\prod_{i=1}^{n} \xi\left(x_{i},\bar{x}_{i}\right)$$

$$=\sum_{\bar{x}_{1}=0}^{1} \cdot...\cdot\sum_{\bar{x}_{n}=0}^{1} \left(1-f_{i}\left(\bar{x}_{1},\ldots,\bar{x}_{n}\right)\right)\cdot\prod_{i=1}^{n} \xi\left(x_{i},\bar{x}_{i}\right)$$

$$=\sum_{\bar{x}_{1}=0}^{1} \cdot...\cdot\sum_{\bar{x}_{n}=0}^{1} (\neg f_{i})\left(\bar{x}_{1},...,\bar{x}_{n}\right)\cdot\prod_{i=1}^{n} \xi\left(x_{i},\bar{x}_{i}\right)=C\left[\neg f_{i}\right]$$

For the last case we consider a binary node with two inputs $f_{i_{1}}(x_{1,1},\ldots,x_{1,n_{1}})$ and $f_{i_{2}}(x_{2,1},\ldots,x_{2,n_{2}})$ which represents a logic gate $\otimes:\{0,1\}^{2}\to\{0,1\}$. We can now show that $C[f_{i_{1}}\otimes f_{i_{2}}]=C[\otimes](C[f_{i_{1}}],C[f_{i_{1}}])$:

$$C\left[\otimes\right](C\left[f_{i_{1}}\right],C\left[f_{i_{2}}\right])=$$

Using the proof for negating nodes we get

and by expanding the product and pulling in the $(\bar{a}_{1}\otimes\bar{a}_{2})$

$$=\sum_{\bar{x}_{1,1}=0}^{1} \cdot...\cdot\sum_{\bar{x}_{2,n_{2}}=0}^{1} (f_{i_{1}}\otimes f_{i_{2}})\left(x_{1,1},\ldots x_{2,n_{2}}\right)\prod_{i=1}^{n_{1}} \xi\left(x_{1,i},\bar{x}_{1,i}\right)\prod_{i=1}^{n_{2}} \xi\left(x_{2,i},\bar{x}_{2,i}\right)$$

$$= C\left[f_{1}\otimes f_{2}\right]$$

To sum up, we can stepwise assemble or calculate the interpolation function by using $C[\neg f]=1-C[f]$ and $C[f_{1}\otimes f_{2}]$ $=C[\otimes](C[f_{1}],C[f_{2}])$.

## Topologies for the benchmarks

The simple topology used to benchmark the simulation speed and the SSS search algorithm has $n$ nodes $x_{1}$ to $x_{n}$ whose Boolean functions are

$$B_{i}=\left\{\begin{matrix} x_{3}\vee...\vee x_{n} & :i\leq2 \\ x_{i} & :3\leq i\leq n/2+1 \\ 0 & :n/2+1<i \end{matrix}\right.$$

This results in a maximum degree of $n-2$, $2.5n-5$ interactions and $2^{(n-2)/2}$stable states.

## Pseudocode for the interpolation algorithm

The following pseudocode gives a concise description of the recursive BooleCube interpolation algorithm. All implementation details and surrounding code, which is a standard implementation of the forth order Runga-Kutta-method, have been omitted for clarity reasons. Only AND and OR binary gates are considered. In the data structures used by the pseudocode, the input nodes of the Boolean tree hold a position information (position(*node*)) which determines which component of the vector of input values (inputValues[]) the node represents. For example, if the input vector was (0, 0.1, 0.9, 0.5), an input node with position = 2 would represent the second component of the vector, i.e. 0.1. The HillCube and normalized HillCube interpolations can by derived from this algorithm by a simple manipulation of the inputValues[] array as defined in [2].

**function** interpolateBooleCube (BooleanTree tree, inputValues[])

**if**(isBinaryGate(root(tree)))

valueOfFirstLeaf = interpolateBooleCube(firstLeaf(root(tree)), inputValues[])

valueOfSecondLeaf = interpolateBooleCube(secondLeaf(root(tree)), inputValues[])

**return** interpolateBinaryGate(root(tree), valueOfFirstLeaf, valueOfSecondLeaf)

**else if**(isNotGate(root(tree)))

**return** 1 - interpolateBooleCube(leaf(root(tree)), inputValues[])

**else**

// root(tree) is an input node

**return** inputValues[position(root(tree))]

**end if**

**end function**

**function** interpolateBinaryGate(BooleanGate gate, firstInput, secondInput)

**if**(gate == AND)

**return** firstInput*secondInput

**else**

// gate == OR

**return** firstInput + secondInput – firstInput*secondInput

**end if**

**end function**

Functions used by the pseudocode:

root() Return the root node of a Boolean tree

isBinaryGate() Return true if the node is a binary Boolean gate

leaf() Returns the subtree rooted in the child node of a unary node in a Boolean tree

firstLeaf() Returns the subtree rooted in the first child node of a binary node in a Boolean tree

secondLeaf() Returns the subtree rooted in the second child node of a binary node in a Boolean tree

isNotGate() Returns true if the node is unary NOT gate

position() Returns the position information of an input node in a Boolean tree
